# *GmNFYA13* Improves Salt and Drought Tolerance in Transgenic Soybean Plants

**DOI:** 10.3389/fpls.2020.587244

**Published:** 2020-10-23

**Authors:** Xiao-Jun Ma, Jin-Dong Fu, Yi-Miao Tang, Tai-Fei Yu, Zhen-Gong Yin, Jun Chen, Yong-Bin Zhou, Ming Chen, Zhao-Shi Xu, You-Zhi Ma

**Affiliations:** ^1^Institute of Crop Science/Institute of Urban Agriculture, Chinese Academy of Agricultural Sciences (CAAS)/National Key Facility for Crop Gene Resources and Genetic Improvement, Key Laboratory of Biology and Genetic Improvement of Triticeae Crops, Ministry of Agriculture, Beijing, China; ^2^Beijing Engineering Research Center for Hybrid Wheat, The Municipal Key Laboratory of the Molecular Genetics of Hybrid Wheat, Beijing Academy of Agriculture and Forestry Sciences, Beijing, China; ^3^Institute of Crop Resources, Heilongjiang Academy of Agricultural Sciences, Heilongjiang, China

**Keywords:** nuclear factor YA, salt and drought tolerance, ABA hypersensitivity, soybean, crop yield

## Abstract

NF-YA transcription factors function in modulating tolerance to abiotic stresses that are serious threats to crop yields. In this study, *GmNFYA13*, an NF-YA gene in soybean, was strongly induced by salt, drought, ABA, and H_2_O_2_, and suppressed by tungstate, an ABA synthesis inhibitor. The *GmNFYA13* transcripts were detected in different tissues in seedling and flowering stages, and the expression levels in roots were highest. GmNFYA13 is a nuclear localization protein with self-activating activity. Transgenic *Arabidopsis* plants overexpressing *GmNFYA13* with higher transcript levels of stress-related genes showed ABA hypersensitivity and enhanced tolerance to salt and drought stresses compared with WT plants. Moreover, overexpression of GmNFYA13 resulted in higher salt and drought tolerance in OE soybean plants, while suppressing it produced the opposite results. In addition, GmNFYA13 could bind to the promoters of *GmSALT3*, *GmMYB84*, *GmNCED3*, and *GmRbohB* to regulate their expression abundance *in vivo*. The data in this study suggested that *GmNFYA13* enhanced salt and drought tolerance in soybean plants.

## Introduction

As types of osmotic stress, salt and drought stresses are major challenges to growth and development of plants, resulting in severe yield losses (Zhu, 2002; Golldack et al., 2014). Many changes related to metabolism and physiology occur when plants are subjected to osmotic stresses (Golldack et al., 2011). These stresses are negatively affecting the available arable land, which is paralleled by the pressing need for food all over the world (Golldack et al., 2011). The signaling pathways of salt and drought stresses have been uncovered in previous researches (Zhu, 2002; Golldack et al., 2011; Lawlor, 2013). Abscisic acid (ABA) is produced in plants under abiotic stresses, especially salt and drought stresses, and the accumulation level of ABA is positively related to salt and drought tolerance of plants (Nakashima and Yamaguchi-Shinozaki, 2013). ABA signaling is complex, including receptors PYR/PYL/RCARs, PP2Cs, SnRK2s, AREB/ABFs, and ABA-responsive genes (Ma et al., 2009; Park et al., 2009; Vlad et al., 2009). Moreover, NCED3 was certified as a key ABA synthetase (Barrero et al., 2006; Hao et al., 2009; Sussmilch et al., 2017; Zhang et al., 2020). Many transcription factors (TFs) were identified to play vital roles in regulating osmotic stress via an ABA-dependent pathway, such as NAC, WRKY, MYB, ERF, and bZIP (Hu et al., 2006; Zhou et al., 2008; Lippold et al., 2009; Peng et al., 2009; Zhang et al., 2009; Amir Hossain et al., 2010; Hsieh et al., 2010; Ren et al., 2010).

NF-Y TFs function in affecting plant growth and development (Kwong et al., 2003; Miyoshi et al., 2003; Xu et al., 2005; Wenkel et al., 2006; Cai et al., 2007; Mu et al., 2008; Wei et al., 2010; Ballif et al., 2011; Stephenson et al., 2011; Hackenberg et al., 2012; Hou et al., 2014; Myers et al., 2016) and are essential in abiotic stress responses. Overexpression of *NFYA5* (Li et al., 2008), *GmNFYA3* (Ni et al., 2013),*GmNFYB1* (Li et al., 2017), and NF-YB2 and NF-YB3 (Sato et al., 2019) conferred drought tolerance to transgenic *Arabidopsis* plants. Transgenic rice plants overexpressing *OsNFYA7* (Lee et al., 2015), *OsHAP2E* (Alam et al., 2015), and *Cdt-NFYC1* (Chen et al., 2015) showed enhanced tolerance to osmotic stresses.

Enhanced tolerance to osmotic stresses is associated with increased transcript levels of stress-related genes. For example, overexpression of *SOS1*, *SOS2*, *SOS3* (Qiu et al., 2002), *GmSALT3* (Guan et al., 2014), *GmSOS1*, and *GmNHX1* (Zhang W. et al., 2019) resulted in enhanced salt tolerance in plants. In addition, *DREB2A* (Nakashima et al., 2000), *ABI3* (Mittal et al., 2014), *RD29A* (Bihmidine et al., 2013), *GmMYB84* (Wang N. et al., 2017), *GmDREB2* (Chen et al., 2007), and *GmWRKY46* (Luo et al., 2013) were vital genes that positively regulated drought tolerance in plants. During osmotic stresses, reactive oxygen species (ROS) are produced and result in oxidative damage to cells, especially cytomembranes (Uchida et al., 2002; Zhou et al., 2018; Cui et al., 2019). A respirator burst oxidase homolog B (RbohB) gene in soybean, *GmRbohB*, was determined to function in ROS scavenging (Wang N. et al., 2017). Therefore, these genes are indispensable for tolerance to salt and drought stresses in plants.

Soybean is an important oil-bearing crop all over the world, and its production is constrained by salt and drought stresses. In this study, overexpression of an NF-Y gene in soybean, *GmNFYA13*, conferred enhanced salt and drought tolerance to transgenic soybean plants, while its abolition resulted in opposite results. Considering these results, *GmNFYA13* should be a candidate gene in molecular breeding of soybean.

## Materials and Methods

### Plant Growth and Treatments

Seeds of the soybean (*Glycine max* L. Merr.) cultivar Williams 82 were grown in plastic pots (14 cm diameter, 15 cm depth) containing nutrient soil and grown for 20 days in a tissue culture room at 28/18°C day/night with a 14-h photoperiod and 70% relative humidity as described previously (Ma et al., 2020), which are considered as normal conditions for soybean plants. The seedlings were used to assess gene expression patterns under several treatments. For H_2_O and salt treatment, the seedlings were subjected to H_2_O and 200 mM NaCl solution for 12 h; for drought treatment, the seedlings were pulled out from soil and placed on a plastic plate for 12 h; for ABA and H_2_O_2_ treatment, the roots were immersed in 100 μM ABA and 10 mM H_2_O_2_ for 12 h. The leaves under various treatments were harvested at 0, 1, 2, 4, 8, and 12 h and frozen at −80°C for isolation of RNA. To test whether an ABA synthesis inhibitor (tungstate) could affect salt-induced *GmNFYA13* transcripts, two detached leaves of soybean plants were placed into ddH_2_O to eliminate the effect of wound stress for 1 h, and then transferred into ddH_2_O and 2 mM tungstate for 3 h, followed by being subjected to 200 mM NaCl solution for 1 h. To determine whether tungstate could suppress the drought-induced expression level of *GmNFYA13*, the “drought + tungstate” treatment was performed as described previously (Ma et al., 2020) and 1 mM naproxen was replaced with 2 mM tungstate. The leaves immersed in ddH_2_O were used as the control. Samples were collected and frozen at −80°C.

To evaluate the transcription levels of *GmNFYA13* in different tissues, the tissues of 20-day-old and 50-day-old soybean plants were collected and stored as described previously (Ma et al., 2020). All of the experiments were performed in a tissue culture room at 28/18°C day/night with a 14-h photoperiod and 70% relative humidity.

### Cloning of *GmNFYA13*

Williams 82 soybean plants were used for RNA isolation, followed by reverse transcription to cDNA. The full-length coding sequence (CDS) of *GmNFYA13* was isolated using PrimerSTAR Max DNA polymerase (Takara, Japan) and primer set 5′-GGTAGTCAATAGTCATCACTT-3′ and 5′-CAAGTTGGATGAGATAAAGCC-3′. The PCR products were cloned into the vector pEASY-Blunt (TransGen, China) and sequenced; the correct clone was stored at −80°C for isolation of the CDS of *GmNFYA13*.

### Generation of Transgenic *Arabidopsis* Lines

The CDS without the termination codon was fused in pCAMBIA1302 vector and ligated with *Nco*I site to generate the pCAMBIA1302:*GmNFYA13* fusion construct, which is driven by the promoter of cauliflower mosaic virus 35S (CaMV35S). The primer set used in this assay was 5′-GGGACTCTTGACCATGATGCCAGGGAAACCT-3′ and 5′-TCAGATCTACCATGGCTTTGAAATCCCCATT-3′. The pCAMBIA1302:*GmNFYA13* fusion constructs were transformed into *Agrobacterium tumefaciens* strain GV3101, followed by infection of *Arabidopsis* flowers. Hygromycin (Roche, Germany) was used to screen the positive transgenic lines to obtain the T_3_ homozygotes. The *35S:GmNFYA13-2*, *35S:GmNFYA13-4*, and *35S:GmNFYA13-5* transgenic *Arabidopsis* lines were used to explore the function of *GmNFYA13*.

### Subcellular Localization

Five-day-old *35S:GmNFYA13* and WT *Arabidopsis* plants on 1/2 MS medium were used to determine the localization of GmNFYA13 in cells. Roots of *35S:GmNFYA13* plants could express GFP-GmNFYA13 fusion protein. Roots of *35S:GmNFYA13* and WT *Arabidopsis* plants were detached and observed using a confocal laser scanning microscope (Zeiss, LSIM700) as described previously (Liu et al., 2013; Ma et al., 2020).

### Promoter:GUS Analysis

The 2000-bp DNA fragment upstream of cDNA was amplified to obtain the *GmNFYA13* promoter using PrimerSTAR Max DNA polymerase (Takara, Japan) with the primer set 5′-GGACACGTGTCTCGATCTTCG-3′ and 5′-CTTAGCACAACTTTTCCCTAT-3′. The products were cloned into a modified pCAMBIA1305 vector to generate the pCAMBIA1305:P*_*GmNFYA13*_* fusion construct with the forward primer 5′-CCATGATTACGAATTCGGACACGTGTCTCGATCTTCG-3′ and reverse primer 5′-CTCAGATCTACCATGGCTTAGCACAA CTTTTCCCTAT-3′ as previously described (Ma et al., 2020). The *A. tumefaciens* strain GV3101 harboring pCAMBIA1305:P*_*GmNFYA13*_* was used to infect *Arabidopsis* flowers. The positive seeds were screened with hygromycin to obtain the T_3_ homozygotes. T_3_-generation transgenic *Arabidopsis* plants were used to carry out further experiments. The P*_*GmNFYA13*_*:GUS analysis was performed as described previously (Jefferson et al., 1987).

To measure the transcripts of GUS in P*_*GmNFYA13*_*:GUS transgenic *Arabidopsis* plants under normal condition, the rosette leaves, cauline leaves, roots, flowers, and siliques were sampled and used for RNA isolation. The special primer set of GUS was listed in Supplementary Table 1.

### Transactivation Activity Assay in Yeast Strains

The full-length CDS of *GmNFYA13* was ligated into the *Nde*I restriction site of the pGBKT7 vector to generate the BD:*GmNFYA13* construct with the forward primer 5′-AGGAGGACCTGCATATGATGCCAGGGAAACCT-3′ and reverse primer 5′-GCCTCCATGGCCATATGTTATTTGAAATC CCC-3′. The construct was transformed into yeast strain AH109. The yeast strains were grown on SD -Trp plates at 30°C for 3 days and then transferred to SD -Trp/-His/-Ade and SD -Trp/-His/-Ade with 4 mg/ml α-Gal. The yeast strains harboring the empty pGBKT7 construct were used as the negative control.

### Germination and Root Growth of *Arabidopsis* Plants

*35S:GmNFYA13* seeds were sterilized as described previously (Ma et al., 2020). For the germination assay, the sterilized seeds were sown on 1/2 MS medium containing 50–80 mM NaCl, 0.5–0.8 μM ABA, and 8–10% PEG 6000 (PEG), followed by stratification for 2 days at 4°C, and then the mediums were transferred in a greenhouse at 22°C with a 16-h photoperiod and 70% relative humidity, which are considered normal conditions for *Arabidopsis* plants. The seeds sown on 1/2 MS medium were set as the control.

For the root growth assay, sterilized seeds were sown on 1/2 MS medium and stratified for 2 days, and then the mediums were transferred into a greenhouse under normal conditions. The 3-day-old seedlings were grown on 1/2 MS medium containing 100–120 mM NaCl, 0.5–0.8 μM ABA, and 10–12% PEG for 7 days, respectively, and then the root lengths were measured using a straightedge.

### Salt and Drought Treatment of *Arabidopsis* Lines

The stratified seeds were grown for 7 days under normal conditions, and then 7-day-old seedlings were transferred into plastic pots (4.5 cm in length and width, 5 cm in depth) for 2 weeks. For salt treatment, the 3-week-old seedlings were irrigated with 350 mM NaCl solution; for drought treatment, water supply was shut off until significant difference was detected between *35S:GmNFYA13* and WT *Arabidopsis* plants, and then the seedlings were watered and allowed to recover for a week.

To assess the transcription levels of salt-responsive genes in the *35S:GmNFYA13* transgenic *Arabidopsis* plants, the leaves of 3-week-old seedlings were collected at 1 h after salt treatment. To assess the transcription levels of drought-related genes in *35S:GmNFYA13* lines, the leaves of 3-week-old seedlings were sampled at 2 h after drought treatment. The collected leaves were immediately frozen with liquid nitrogen for RNA isolation. The leaves of 3-week-old seedlings under normal conditions were set as the control.

### Generation of Transgenic Soybean Plants

To generate transgenic soybean plants overexpressing *GmNFYA13*, the CDS of *GmNFYA13* without the termination codon was introduced into the vector pCAMBIA3301 to generate the pCAMBIA3301:*GmNFYA13* construct driven by the CaMV35S promoter and ligated with *Nco*I and *Bst*EII sites with the primer set 5′-GGACTCTTGACCATGATGCCAGGGAAACCTGAC-3′ and 5′-ATTCGAGCTGGTCACCTTTGAAATCCCCATTAGG-3′. To generate transgenic soybean plants suppressing *GmNFYA13*, a 705-bp RNA interference fragment (Supplementary Figure 1) was artificially synthesized and inserted into the same vector to generate the pCAMBIA3301:RNAi-*GmNFYA13* construct. The pCAMBIA3301:*GmNFYA13* (OE), pCAMBIA3301:RNAi-*GmNFYA13* (RNAi), and pCAMBIA3301 empty vector (EV) were transformed into *A. rhizogenes* K599 and stored at −80°C.

Soybean seeds were sown into nutrient soil in plastic pots (14 cm diameter, 15 cm depth) and the hypocotyls of 5-day-old seedlings were infected by *A. rhizogenes* K599 harboring OE, RNAi, and EV construct with a syringe tip, and then covered by plastic cups for 24 h without light. The soybean plants were uncovered, and the infected hypocotyls were surrounded by peat and vermiculite mixture (1:1, v/v). They were irrigated with enough water to create a warm and humid environment for 2 weeks, and then hairy roots were induced. The principal roots were cut off, and soybean plants with positive hairy roots screened by QuickStix kit (EnviroLogix, America) and qRT-PCR were used to perform subsequent experiments; they were, respectively, named OE, RNAi, and EV transgenic soybean plants.

### Salt and Drought Treatments of Transgenic Soybean Plants

The transgenic soybean plants with positive hairy roots were planted in plastic pots (14 cm diameter, 15 cm depth) containing nutrient soil for 7 days to establish normal growth. It is difficult to complete the entire growth period using plants with only a few positive hairy roots under salt and drought stresses. However, the transgenic soybean plants are sufficient for drought tolerance at the seedling stage.

For salt treatment, the soybean plants were irrigated with 500 mM NaCl solution; for drought treatment, the water supply to soybean plants was shut off until significant difference was observed among the three transgenic soybean lines.

To evaluate the transcription level of salt/drought-related genes in transgenic soybean plants, for salt and drought treatments, the positive hairy roots were sampled at 1 h after salt stress and 2 h after drought stress. The control was the non-treated positive hairy roots.

### Quantitative Real-Time PCR (qRT-PCR) Analysis

The qRT-PCR analysis was performed using an Applied Biosystems 7500 real-time PCR system and the data were analyzed with the 2^–ΔΔ*CT*^ method based on C_T_ values (Ni et al., 2013). The specific primers for qRT-PCR in this study are in Supplementary Table 1.

### Measurement of Water Loss, Soil Water Potential (SWP), and Proline Content

In *35S:GmNFYA13 Arabidopsis* and three transgenic soybean lines, water loss and SWP were measured as described previously (Li et al., 2008; Ma et al., 2020). The proline content was measured as described previously (Cui et al., 2019) after drought treatment for 4 days and 5 days with respect to *35S:GmNFYA13 Arabidopsis* and transgenic soybean plants, respectively.

### Measurement of Relative Water Content (RWC), Malondialdehyde (MDA), Chlorophyll, and Ion Leakage

In *35S:GmNFYA13 Arabidopsis* and three transgenic soybean lines, RWC, MDA, chlorophyll, and ion leakage were measured after drought and salt treatment as described previously (Ni et al., 2013; Ma et al., 2020).

### Measurement of ABA Concentration and Stomatal Aperture

Abscisic acid concentration was measured when *35S:GmNFYA13* transgenic *Arabidopsis* plants were subjected to salt and drought treatment for 4 days as described previously (Wang et al., 2018). Leaves of 3-week-old *35S:GmNFYA13* plants were detached and immersed into stomatal opening buffer, which was prepared for 3 h as previously reported (Tian et al., 2015); leaves were then transferred to 0, 5, and 10 μM ABA solution for 2 h. Stomatal apertures were detected using a confocal laser scanning microscope (Zeiss, LSIM700) and measured with the ruler tool in Adobe Photoshop CS5.

### Transcriptional Activation Assay in Tobacco Plants

Tobacco seeds were sterilized with 1% sodium hypochlorite for 15 min, washed three times using sterile water, and then sown on 1/2 MS medium. Seven-day-old seedlings were transferred into nutrient soil in a greenhouse at 25/22°C (day/night) with a 16-h photoperiod and 50–60% relative humidity as described previously (Zhang et al., 2017). Tobacco plants with four leaves were infected.

The promotors of target genes were inserted into pGreen II 0800 as previously described (Ma et al., 2020). The primer set for *GmSALT3* was 5′-GTCGACGGTATCGATAAGCTTCGATGATACTACATAAGT-3′ and 5′-CAGGAATTCGATATCAAGCTTGGCCAAAGACTC AGTGCT-3′, with 5′-GTCGACGGTATCGATAAGCTTCCTGG TCCACTCTAGTAG-3′ and 5′-CAGGAATTCGATATCAAGCT TGGTGAATGGTCGGTTGAA-3′ for *GmNCED3*. Primers for *GmMYB84* were 5′-GTCGACGGTATCGATAAGCTTCTCAG CTTCAATTACAAT-3′ and 5′-CAGGAATTCGATATCAAGCT TGGCTATTCTCACTCACTA-3′; primers for *GmRbohB* were 5′-GTCGACGGTATCGATAAGCTTACGACACAGTCTGAGAAT-3′ and 5′-CAGGAATTCGATATCAAGCTTCTCCTCGTCGTCC TTGAA-3′. The fusion constructs pGreen II 0800:P*_*GmSALT3*_*_/_*_*GmNCED3*_*_/_*_*GmMYB84*_*_/_*_*GmRbohB*_* were transformed into *A. tumefaciens* strain GV3101. GV3101 harboring the pCAMBIA1302 empty vector and pCAMBIA1302:*GmNFYA13* fusion construct were co-transformed into tobacco leaves with GV3101 harboring pGreen II 0800:P*_*GmSALT3*_*_/_*_*GmNCED3*_*_/_*_*GmMYB84*_*_/_*_*GmRbohB*_*. The GUS fluorescence was observed using LB985 NightSHADE (Berthold, Germany) following the manufacturer’s instructions.

To quantify the LUC activity in the tobacco leaves of equal weight, the protein was extracted. The extraction buffer consisted of 50 mM Tris, 0.5 M sucrose, 1 mM MgCl_2_, 10 mM EDTA, and 5 mM DTT, and then pH was adjusted to 8.0. The LUC expression levels of the protein solution were evaluated with GloMax Multi Jr (Promega, America) according to the manufacturer’s instructions.

### Statistical Analysis

The data in this research were subjected to Duncan’s tests using SPSS Statistics 22 (IBM, United States). The data of germination rate, water loss, and SWP were assessed with Student’s *t*-test. Three biological replications were performed for each experiment in this study.

## Results

### Expression Pattern of NF-YA Genes in Soybean

**FIGURE 1:**
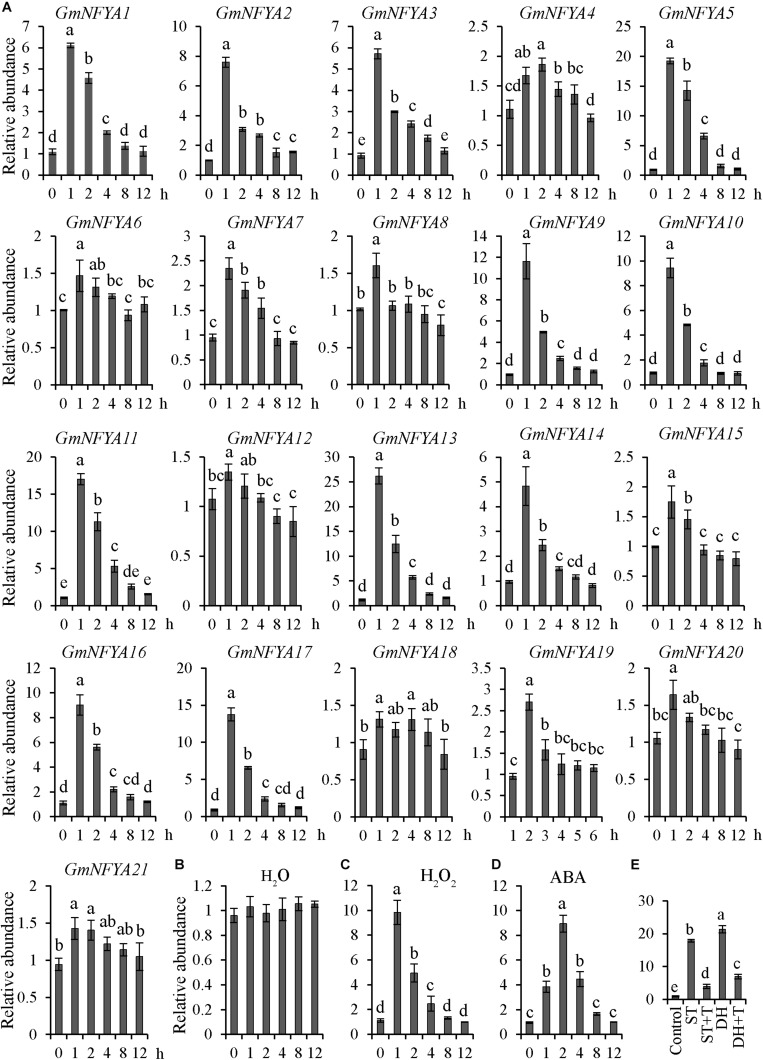
*GmNFYA13* transcripts were induced by treatment of salt, H_2_O_2_, and ABA. **(A)** The expression levels of 21 NF-YA members in *Glycine max* were evaluated with qRT-PCR under salt treatment. **(B–D)** H_2_O, H_2_O_2_, and ABA induced *GmNFYA13* transcripts. **(E)** Tungstate suppressed salt- and drought-induced *GmNFYA13* transcripts. Control, salt, salt + tungstate, drought, and drought + tungstate are indicated by CTR, ST, ST + T, DH, and DH + T, respectively. *GmCYP2* was used as the internal control and transcripts of all genes used in the assay were normalized to the non-treated expression level, which was set as 1.0. Data indicate three biological replicates ± SD. Different letters above the columns represent significant differences at *P* < 0.05.

As described previously, the transcription level of *GmNFYA5* was the highest among all 21 *GmNFYAs* under drought conditions (Ma et al., 2020). To determine whether *GmNFYAs* were induced by salt treatment, the transcription levels of 21 *GmNFYAs* were analyzed by qRT-PCR. The results showed that 12 members were highly responsive to salt stress and the transcript level of *GmNFYA13* was the highest (Figure 1A). In addition, the *GmNFYA13* transcript was higher than other *GmNFYAs* except *GmNFYA5* and *GmNFYA11* under drought treatment (Ma et al., 2020). Moreover, the transcript level of *GmNFYA13* was induced by H_2_O_2_ and ABA treatment (Figures 1C,D) but not by H_2_O (Figure 1B). To further explore whether ABA was involved in salt- and drought-induced *GmNFYA13* transcription, detached leaves were subjected to tungstate solution, which is an ABA biosynthesis inhibitor, followed by salt treatment for 1 h, or drought treatment for 2 h. Compared with the control condition, the *GmNFYA13* transcript under salt/drought treatment was similar to the above expression pattern (Figure 1E). However, pretreatment with tungstate suppressed the induction of *GmNFYA13* expression (Figure 1E). These results showed that ABA played an essential role in salt- and drought-induced *GmNFYA13* transcription.

The coding sequence of *GmNFYA13* (Glyma12g36540) is 912 bp in length, encoding a 34.06-KD polypeptide with an isoelectric point (p*I*) of 9.50. The conserved core regions of GmNFYA13, which consists of an NF-YB/C binding subdomain and CCAAT binding sequences, are similar to those of *Arabidopsis* (Supplementary Figure 2A). Phylogenetic analysis of conserved amino acid sequences showed that GmNFYA13 clustered with AtNFYA2 and AtNFYA10 in *Arabidopsis* (Supplementary Figure 2B).

### Tissue-Specific Expression Analysis and Characterization of GmNFYA13

**FIGURE 2:**
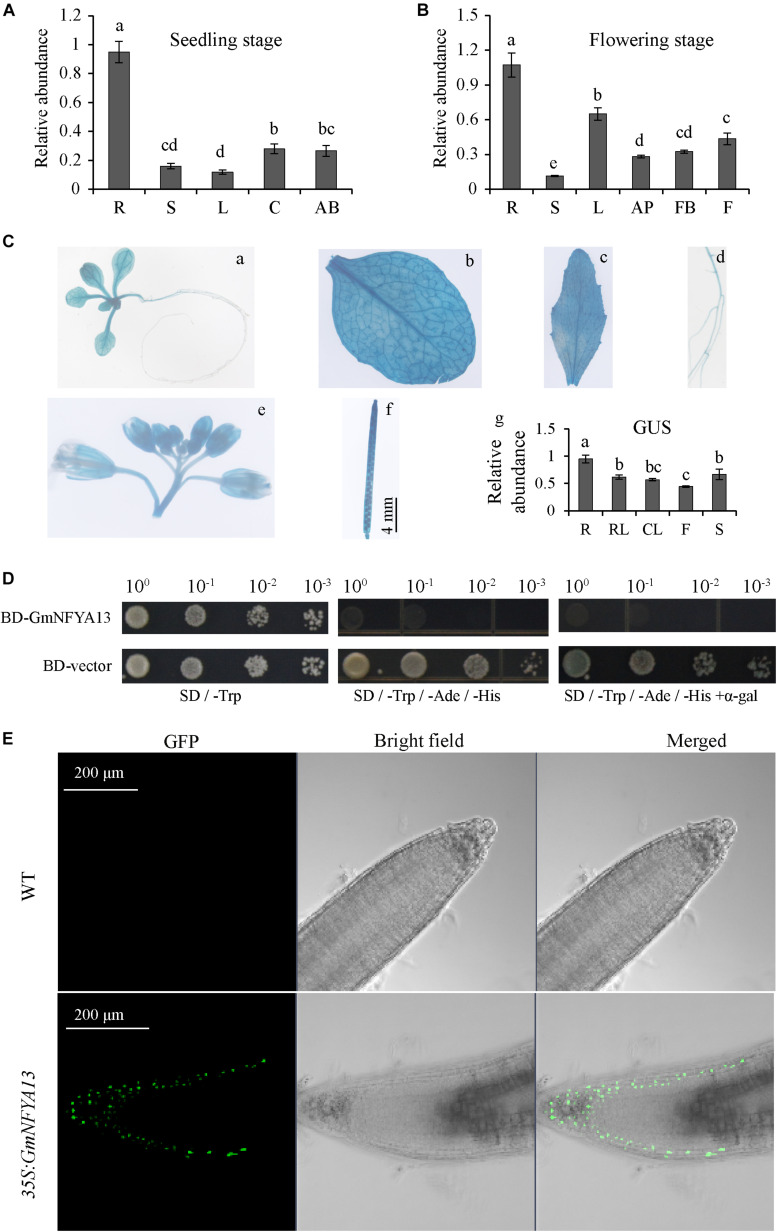
Analysis of tissue-specific expression, self-activating activity, and subcellular localization of GmNFYA13. **(A,B)** *GmNFYA13* transcripts were assessed in various tissues at two growth periods. The expression level of *GmNFYA13* in roots was set as 1.0, and *GmCYP2* was the internal control. Data indicate three biological replicates ± SD. Different letters above the columns represent significant differences at *P* < 0.05. **(C)** GUS staining in each tissue in P_*GmNFYA13*_:GUS transgenic *Arabidopsis* plants. *a*, 5-day-old seedling; *b*, rosette leaf (RL); *c*, cauline leaf (CL); *d*, root (R); *e*, flower (F); *f*, silique (S); *g*, transcripts of GUS were measured in various tissues of transgenic *Arabidopsis* by qRT-PCR. Scale bar = 4 mm. The transcripts of GUS in roots were set as 1.0, and *AtTub8* was the internal control. Data indicate three biological replicates ± SD. Different letters above the columns represent significant differences at *P* < 0.05. **(D)** Self-activating activity analysis of GmNFYA13. **(E)** GFP fluorescence was detected in roots of WT and *35S:GmNFYA13 Arabidopsis* plants. Scale bar = 200 μm. Each experiment had three biological replicates.

The transcription of *GmNFYA13* was detected in various tissues at seedling and flowering growth phases based on qRT-PCR (Figures 2A,B). The *GmNFYA13* transcript was highest in roots in both stages and significantly improved in leaves of the former period compared with that of the latter (Figures 2A,B). To better evaluate the tissue-specific expression of *GmNFYA13*, T_3_-generation transgenic *Arabidopsis* plants overexpressing P*_*GmNFYA13*_*:GUS were generated. GUS staining was observed in *Arabidopsis* seedling, rosette leaf, cauline leaf, root, flower, and silique (Figure 2C). The GUS expression level in leaves was lower than that in roots (Figure 2C), consistent with the data of qRT-PCR in soybean plants.

To test whether GmNFYA13 independently regulated downstream genes, the self-activating activity was evaluated in yeast strains. The yeast strains harboring BD:*GmNFYA13* fusion construct could grow on SD -Trp/-His/-Ade and SD -Trp/-His/-Ade with α-Gal, where no yeast strains harboring BD empty vector could grow. The results implied that GmNFYA13 had self-activating activity (Figure 2D).

To observe the localization of GmNFYA13 in plant cells, roots of WT and *35S:GmNFYA13 Arabidopsis* plants were detached. The GFP fluorescence in roots of *35S:GmNFYA13* plants was exclusively detected in the nuclei with no fluorescence observed in roots of WT plants (Figure 2E), which showed that GmNFYA13 was a nuclear localization protein.

### Sensitivity of *35S:GmNFYA13 Arabidopsis* Plants to ABA, NaCl, and PEG

**FIGURE 3:**
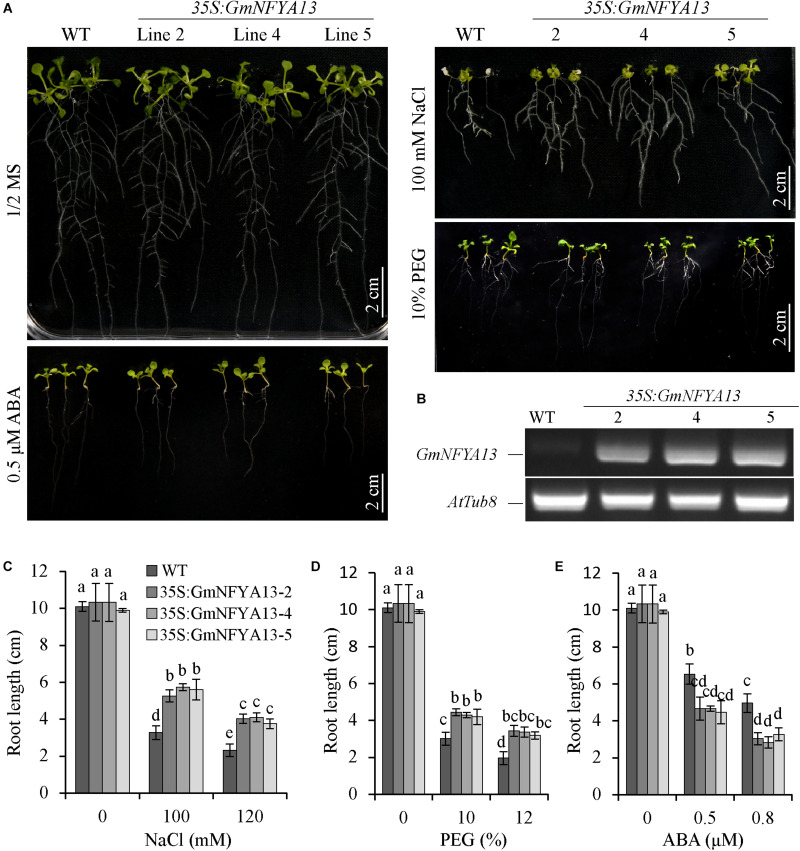
Root growth of WT and *35S:GmNFYA13* transgenic *Arabidopsis* seedlings under treatments of salt, drought, and ABA. **(A)** The root elongation of WT and *35S:GmNFYA13* with/without treatments. Scale bar = 2 cm. **(B)** The transcript levels of *GmNFYA13* in WT and *35S:GmNFYA13* lines were evaluated with RT-PCR. *AtTub8* was the internal control. **(C–E)** The root lengths of WT and *35S:GmNFYA13* lines subjected to various concentrations of NaCl, PEG, and ABA. Data indicate three biological replicates (*n* = 18) ± SD. Different letters above the columns represent significant differences at *P* < 0.05. Three biological replicates were used in each experiment.

Three *35S:GmNFYA13 Arabidopsis* lines (*35S:GmNFYA13-2*, *35S:GmNFYA13-4*, and *35S:GmNFYA13-5*) were used to carry out further assays based on RT-PCR analysis (Figure 3B and Supplementary Figure 5). In the germination assay, there was no significant difference between *35S:GmNFYA13* and WT *Arabidopsis* seeds with respect to germination rates under control conditions (Supplementary Figure 3A). However, in comparison to WT *Arabidopsis* seeds, the germination rate of *35S:GmNFYA13* seeds was higher when subjected to different concentrations of NaCl and PEG, but lower under treatment of ABA (Supplementary Figures 3B–G).

In the root elongation assay, there was no significant difference between root lengths of *35S:GmNFYA13* and WT seedlings prior to these three treatments (Figure 3A). Consistent with the germination assay results, under treatments of salt and PEG, the root elongation of WT seedlings was more severely suppressed in comparison to that of *35S:GmNFYA13* lines (Figures 3A,C,D). Under ABA treatments, the root length of WT seedlings was significantly longer than that of *35S:GmNFYA13* seedlings (Figures 3A,E).

### Tolerance of *35S:GmNFYA13 Arabidopsis* Lines to Salt Treatment

**FIGURE 4:**
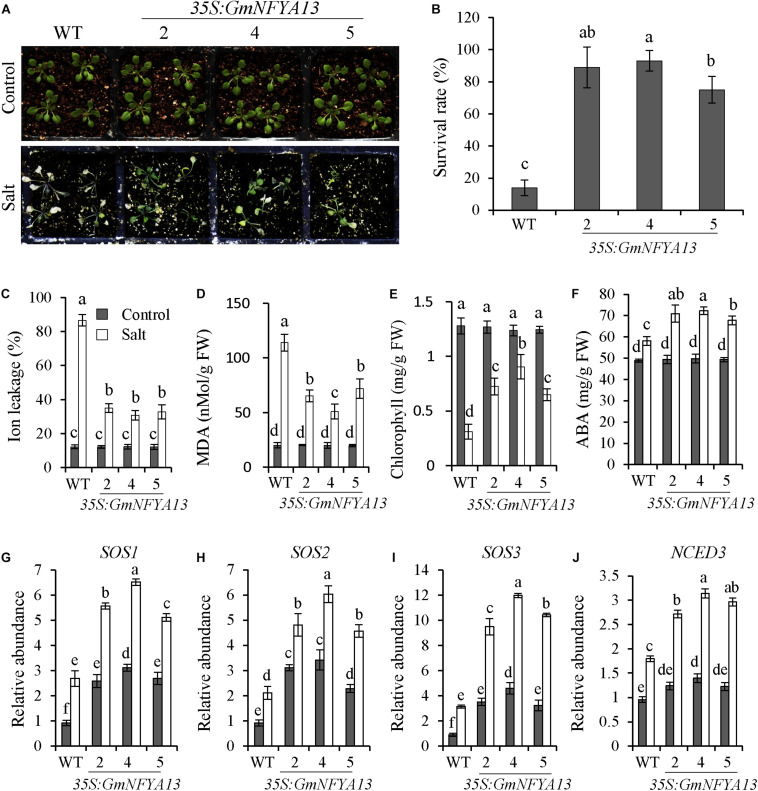
*35S:GmNFYA13 Arabidopsis* seedlings showed enhanced salt tolerance compared to WT lines. **(A)** Assessment of salt tolerance in WT and *35S:GmNFYA13* transgenic *Arabidopsis* plants. Three-week-old seedlings were treated with 350 mM NaCl solution for 7 days. **(B–E)** The survival rate, ion leakage, MDA, and chlorophyll content of WT and *35S:GmNFYA13* lines were measured after salt treatment. **(F)** The ABA concentration of WT and *35S:GmNFYA13* lines were measured following salt treatment for 4 days. **(G–J)** The expression levels of *SOS1*, *SOS2*, *SOS3*, and *NCED3* in WT and *35S:GmNFYA13* lines were evaluated under control and salt treatments. Transcript levels of these genes were normalized to those in WT plants under control conditions, and *AtTub8* was used as the internal control. Data indicate three biological replicates ± SD. Different letters above the columns represent significant differences at *P* < 0.05. Each experiment had three biological replicates.

To elucidate the function of *GmNFYA13* in regulating salt tolerance of *Arabidopsis* plants, 3-week-old *35S:GmNFYA13* and WT plants were subjected to 350 mM NaCl solution for 7 days. Leaves of WT plants were bleached, whereas most leaves from *35S:GmNFYA13* plants remained green with no significant difference observed between the former and latter sets of plants under control conditions (Figure 4A). Consistently, compared with *35S:GmNFYA13* plants, the survival rate and chlorophyll content were lower, and the ion leakage and MDA contents in WT plants were apparently higher (Figures 4B–E). No significant difference was noted between *35S:GmNFYA13* and WT *Arabidopsis* lines with respect to physiological indices under control conditions (Figures 4B–E).

To determine how *GmNFYA13* regulated salt tolerance of *35S:GmNFYA13 Arabidopsis* plants, salt-related genes were analyzed in *35S:GmNFYA13* and WT lines by qRT-PCR under control and salt treatments. Under control conditions, transcript abundance of *SOS1*, *SOS2*, and *SOS3* in *35S:GmNFYA13* lines were higher than those in WT lines with no significant difference detected for the *NCED3* transcript. However, under salt conditions, transcript levels of *SOS1*, *SOS2*, *SOS3*, and *NCED3* in *35S:GmNFYA13* plants were markedly higher compared with WT plants (Figures 4G–J).

### Tolerance of *35S:GmNFYA5 Arabidopsis* Lines to Drought Treatment

**FIGURE 5:**
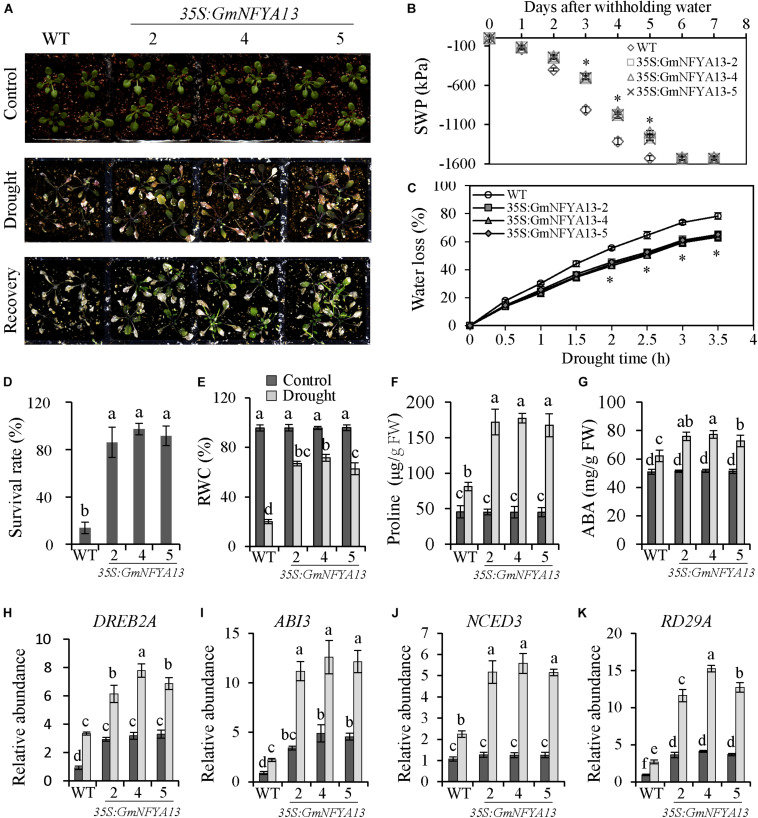
*35S:GmNFYA13 Arabidopsis* seedlings showed enhanced drought tolerance compared to WT lines at the seedling stage. **(A)** Evaluation of drought tolerance in WT and *35S:GmNFYA13* transgenic *Arabidopsis* plants. Three-week-old seedlings were subjected to drought treatment for 9 days and recovered for 7 days. **(B)** The SWP of the soil where WT and *35S:GmNFYA13* were grown. **(C)** The water loss of detached leaves from WT and *35S:GmNFYA13 Arabidopsis* seedlings. **(D)** The survival rate of WT and *35S:GmNFYA13 Arabidopsis* seedlings after recovery from drought treatment. **(E,F)** The relative water content and proline content of WT and *35S:GmNFYA13 Arabidopsis* seedlings after drought treatment. **(G)** The ABA concentration of WT and *35S:GmNFYA13 Arabidopsis* seedlings following drought treatment for 4 days. **(H–K)** Transcript levels of *DREB2A*, *ABI3*, *NCED3*, and *RD29A* under control and drought treatment. Transcript levels of these genes were normalized to those in WT plants under control conditions, *AtTub8* was used as the internal control. Data indicate three biological replicates ± SD. Different letters above the columns represent significant differences at *P* < 0.05. Three biological replicates were used in each experiment.

To evaluate the role of *GmNFYA13* in drought tolerance, water supply was shut off for 9 days. More extreme wilting was observed in WT seedlings compared with *35S:GmNFYA13* seedlings with no difference detected under control conditions (Figure 5A). During drought treatment, the SWP was measured. Compared with WT lines, the SWP of *35S:GmNFYA13* lines fell more slowly, and the minimum level was reached at 5 and 6 days after drought treatment with respect to the former and latter sets of seedlings (Figure 5B). Consistently, in comparison to WT lines, detached leaves in *35S:GmNFYA13* lines lost water more slowly (Figure 5C). The survival rate and RWC of WT lines was lower compared with that of *35S:GmNFYA13* lines (Figures 5D,E). The proline and ABA contents were measured at 4 days after drought stress, and proline content was higher in *35S:GmNFYA13* lines compared with WT lines (Figure 5F).

In addition, ABA was produced in *Arabidopsis* plants treated with drought and salt stresses and ABA accumulation in *35S:GmNFYA13* lines was higher than WT lines (Figures 4F, 5G). ABA triggers stomatal pore closure in leaves (Nakashima and Yamaguchi-Shinozaki, 2013), so the stomatal apertures were observed and measured. When treated with different concentrations of ABA, the stomatal apertures of detached leaves in WT seedlings were significantly bigger than those in *35S:GmNFYA13* seedlings with no difference noted under control conditions (Supplementary Figures 4A,B).

To further understand how *GmNFYA13* functioned in regulating drought tolerance of *35S:GmNFYA13 Arabidopsis* plants, the drought-responsive genes were evaluated by qRT-PCR. Under control and drought conditions, expression levels of *DREB2A*, *ABI3*, *RD29A*, and *NCED3* in *35S:GmNFYA13 Arabidopsis* plants were higher than those in WT plants with no significant difference detected for the *NCED3* transcript under control conditions (Figures 5H–K).

### Tolerance of Transgenic Soybean Plants to Salt Treatment

**FIGURE 6:**
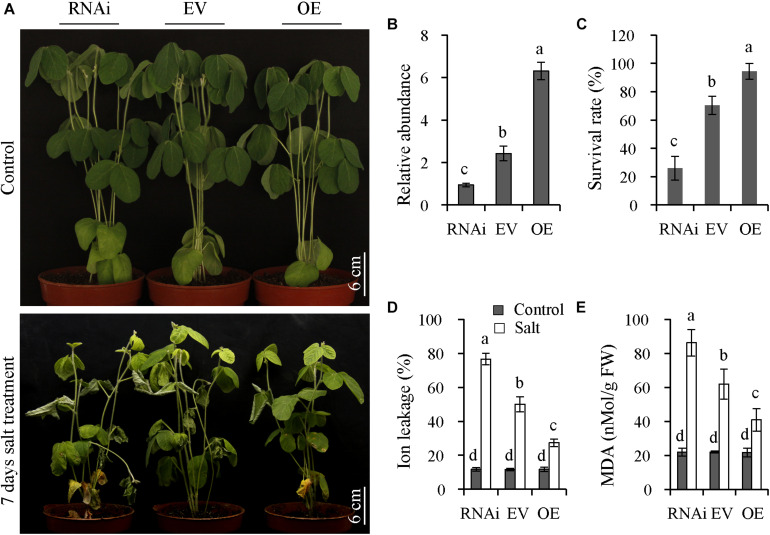
OE and RNAi transgenic soybean plants, respectively, displayed improved and decreased salt tolerance compared with EV plants. **(A)** Assessment of salt tolerance in RNAi, EV, and OE transgenic soybean plants. Soybean plants with positive hairy roots were subjected to 500 mM NaCl solution for 7 days. Scale bar = 6 cm. **(B)** *GmNFYA13* transcript was evaluated in RNAi, EV and OE transgenic soybean plants. *GmCYP2* was used as the internal control and transcripts of *GmNFYA13* were normalized to the expression level in RNAi transgenic soybean plants, which was set as 1.0. Data indicate three biological replicates ± SD. Different letters above the columns represent significant differences at *P* < 0.05. **(C–E)** The survival rate, ion leakage, and MDA content in RNAi, EV, and OE transgenic soybean plants. Data indicate three biological replicates (*n* = 18) ± SD. Different letters above the columns represent significant differences at *P* < 0.05.

To explore the functions of *GmNFYA13*, OE, RNAi, and EV transgenic soybean lines with positive hairy roots were generated. Compared with EV lines, the expression levels of *GmNFYA13* in OE and RNAi lines were significantly higher and lower, respectively (Figure 6B). The transgenic soybean lines were subjected to 500 mM NaCl solution for 7 days. Under salt treatment, the RNAi plants showed chlorosis, and the EV plants displayed less damage than RNAi lines with turgor maintained in OE plants (Figure 6A); the survival rate of OE plants overexpressing *GmNFYA13* was significantly higher than EV plants, while its abolition resulted in the opposite effect (Figure 6C); compared with EV plants, the ion leakage and MDA content in OE and RNAi plants were significantly lower and higher, respectively (Figures 6D,E). However, no significant difference was detected among these indices in OE, EV, and RNAi plants under control conditions (Figures 6C–E).

### Tolerance of Transgenic Soybean Plants to Drought Treatment

**FIGURE 7:**
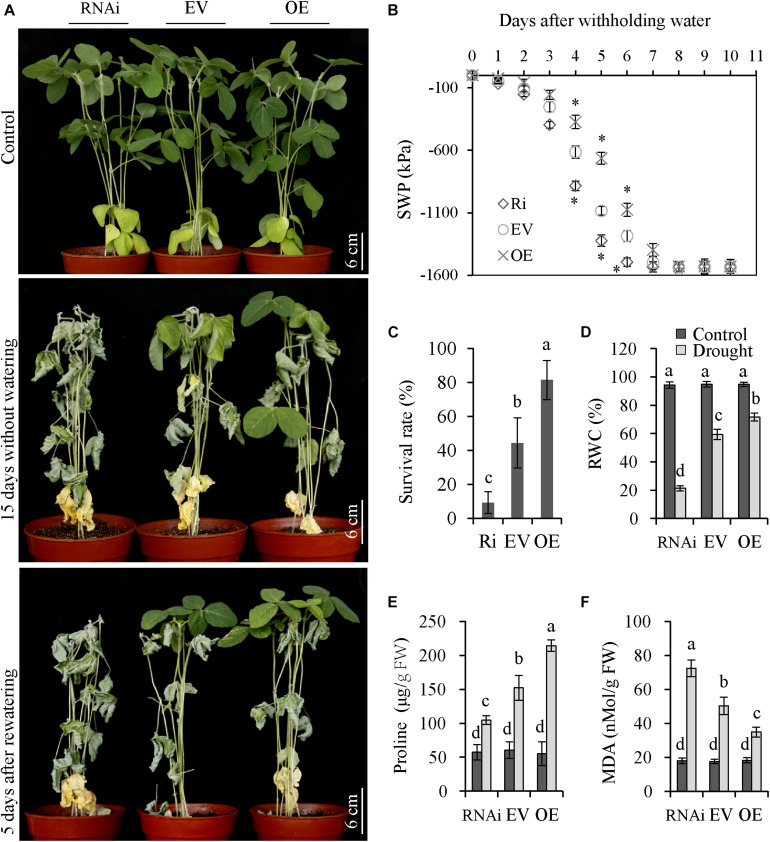
OE and RNAi transgenic soybean plants, respectively, displayed increased and decreased tolerance to drought stress compared with EV plants. **(A)** Drought tolerances were evaluated in RNAi, EV, and OE transgenic soybean plants. Soybean plants were subjected to drought treatment for 15 days and recovery for 5 days. Scale bar = 6 cm. **(B)** The SWP of the soil where RNAi, EV, and OE transgenic plants grew were assessed. Asterisks represent significant differences at *P* < 0.05 in comparison to the corresponding controls. **(C)** The survival rate was measured in RNAi, EV, and OE transgenic plants after recovery from drought treatment. **(D–F)** The relative water content, proline, and MDA content were measured in RNAi, EV, and OE transgenic plants after drought treatment. Data indicate three biological replicates (*n* = 18) ± SD. Different letters above the columns represent significant differences at *P* < 0.05. Each experiment had three biological replicates.

To assess the role of *GmNFYA13* in drought tolerance, OE, RNAi, and EV transgenic soybean lines were deprived of water for 15 days. The OE plants displayed less wilting compared with EV plants, while RNAi plants showed the opposite results (Figure 7A). During drought treatment, the SWP of the soil was measured. After initiation of drought stress, SWP of OE, EV, and RNAi lines declined to the minimum levels at 8, 7, and 6 days, respectively (Figure 7B).

Compared with EV plants, the survival rate, RWC, and proline content of OE and RNAi plants were significantly higher and lower, respectively (Figures 7C–E). The OE plants overexpressing *GmNFYA13* produced significantly lower MDA content, while RNAi plants where it was abolished showed the opposite results (Figure 7F). No significant difference was noted among physiological indices of OE, RNAi, and EV plants under control conditions (Figures 7C–F).

### Transcript Levels of Salt/Drought-Responsive Genes

To investigate how *GmNFYA13* functioned in salt/drought tolerance in transgenic soybean plants, three salt-related genes (*GmSALT3*, *GmSOS1*, and *GmNHX1*) (Guan et al., 2014; Zhang W. et al., 2019), one key gene involved in ABA synthesis (*GmNCED3*) (Zhang X. Z. et al., 2019), three drought-responsive genes (*GmDREB1*, *GmWRKY46* and *GmMYB84*) (Chen et al., 2007; Luo et al., 2013; Wang N. et al., 2017; Ma et al., 2020), and one gene, *GmRbohB*, related to oxidative stress (Wang N. et al., 2017), were selected for further analysis.

**FIGURE 8:**
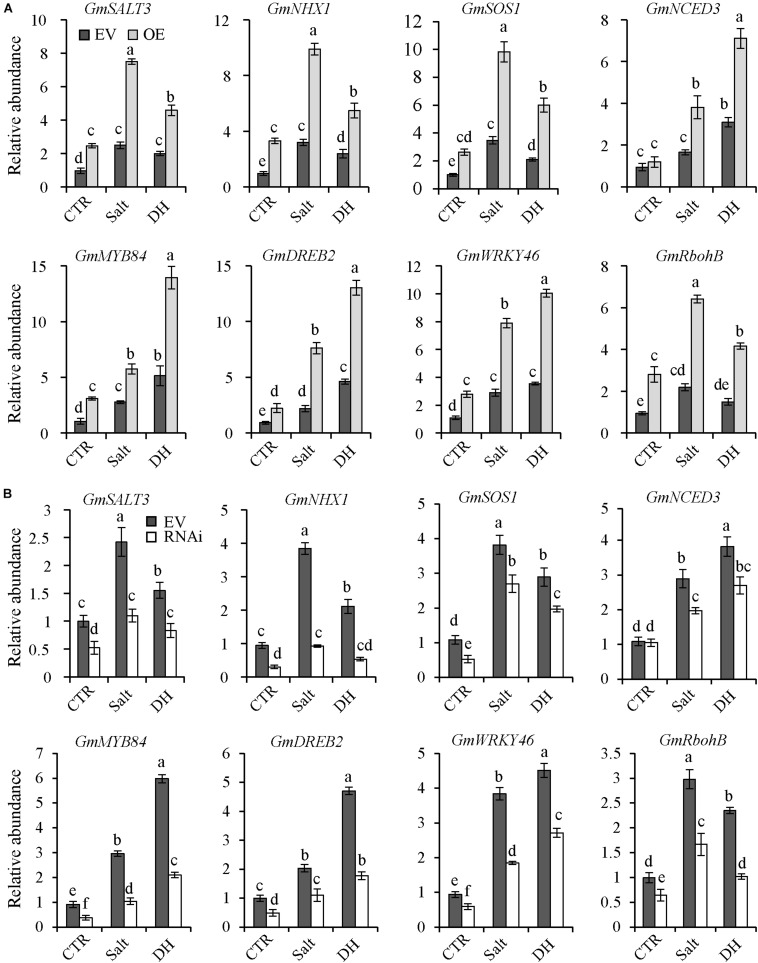
The expression levels of *GmSALT3*, *GmNHX1*, *GmSOS1*, *GmNCED3*, *GmMYB84*, *GmDREB2*, *GmWRKY46*, and *GmRbohB* in RNAi, EV, and OE transgenic soybean plants under control, salt, and drought conditions. **(A)** Transcripts of *GmSALT3*, *GmNHX1*, *GmSOS1*, *GmNCED3*, *GmMYB84*, *GmDREB2*, *GmWRKY46*, and *GmRbohB* in EV and OE transgenic lines under control, salt, and drought conditions. **(B)** Transcript levels of these genes in RNAi and EV transgenic lines under control, salt, and drought conditions. *GmCYP2* was used as the internal control and transcripts of *GmNFYA13* were normalized to the expression level in EV transgenic soybean plants under control conditions, which was set as 1.0. CTR represents the control condition, and salt and DH represent salt and drought treatment, respectively. For salt treatment, roots were subjected to 500 mM NaCl solution for 1 h; for drought treatment, roots were placed on a plastic plate for 2 h; roots under normal condition were used as the control sample. Data indicate three biological replicates ± SD. Different letters above the columns represent significant differences at *P* < 0.05.

Under control conditions, no significant difference was detectable for transcript abundance of *GmNCED3* among OE, EV, and RNAi soybean plants (Figures 8A,B); transcript levels of *GmSALT3*, *GmNHX1*, *GmSOS1*, *GmDREB1*, *GmWRKY46*, *GmMYB84*, and *GmRbohB* in OE plants were higher compared with EV plants (Figure 8A); the opposite results were detected in RNAi plants (Figure 8B). Under salt and drought treatments, compared with WT plants, expression levels of all genes in OE plants were significantly higher and those in RNAi plants were significantly lower (Figures 8A,B).

### Transcriptional Activation Assays

**FIGURE 9:**
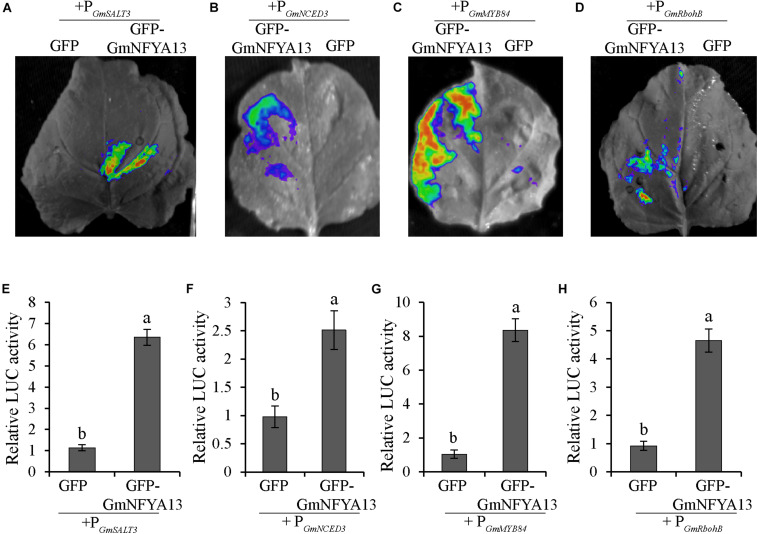
LUC activity was enhanced due to the regulation of GmNFYA13 on promoters of *GmSALT3*, *GmNCED3*, *GmMYB84*, and *GmRbohB*. **(A–D)** The LUC activity was evaluated in tobacco leaves with LB985 NightSHADE. **(E–H)** The LUC expression levels of the protein solution extracted from the leaves, indicated by ratio of LUC/REN, were measured with GloMax Multi Jr. The ratio of LUC/REN of GFP-GmNFYA13 + P*_*GmSALT3*_*_/_*_*GmNCED3*_*_/_*_*GmMYB84*_*_/_*_*GmRbohB*_* was normalized to that of GFP + P*_*GmSALT3*_*_/_*_*GmNCED3*_*_/_*_*GmMYB84*_*_/_*_*GmRbohB*_*, which was set as 1.0. Data indicate three biological replicates ± SD. Different letters above the columns represent significant differences at *P* < 0.05.

To test whether GmNFYA13 could directly regulate the downstream genes *in vivo*, LUC activity in the tobacco leaves harboring recombinant pCAMBIA1302:*GmNFYA13* or pCAMBIA1302 empty vector in consort with pGreen II 0800:P*_*GmSALT3*_*_/_*_*GmNCED3*_*_/_*_*GmMYB84*_*_/_*_*GmRbohB*_* were assessed. Consistent with our expectation, the LUC expression levels in the former sets of leaves were significantly higher than those in the latter sets, which showed that GmNFYA13 could positively modulate the expression levels of *GmSALT3*, *GmMYB84*, *GmNCED3*, and *GmRbohB* by binding to their promotors (Figures 9A–H).

## Discussion

Compared with *Arabidopsis* and rice plants, the soybean genome is larger, containing 115 NF-Y genes and 21 NF-YA members (Thao and Tran, 2012). However, only *GmNFYA3* (Ni et al., 2013) and *GmNFYA5* (Ma et al., 2020) had been functionally characterized. In addition, overexpression of *GmNFYA3* and *GmNFYA5* could only confer drought tolerance to transgenic plants (Ni et al., 2013; Ma et al., 2020). In this study, transgenic *Arabidopsis* and soybean plants carrying extra copies of *GmNFYA13* displayed improved salt and drought tolerance.

The *GmNFYA13* transcript was highest among 21 NF-YA genes in soybean following salt treatment (Figure 1A) and higher than other NF-YA genes except *GmNFYA5* and *GmNFYA11* when subjected to drought treatment (Ma et al., 2020). In addition, the expression level of *GmNFYA13* was highest in roots compared with other tissues (Figure 2A). Likewise, *GmNFYA3*, *AtNFYA5*, and *Cdt-NFYC1* (Li et al., 2008; Ni et al., 2013; Chen et al., 2015), which were induced by osmotic stresses with high levels in roots, functioned in modulating salt or drought tolerance of plants. These results implied that *GmNFYA13* could play important roles in regulating salt and drought tolerance in plants.

In addition, *GmNFYA13* was induced by ABA treatment (Figure 1D), and pretreatment with tungstate suppressed the salt- and drought-induced *GmNFYA13* transcript (Figure 1E), showing that ABA was involved in response to osmotic stresses of *GmNFYA13*. ABA is a hormonal molecule produced during drought and salt stresses (Zhu, 2002; Barrero et al., 2006; Nakashima and Yamaguchi-Shinozaki, 2013). Under salt and drought treatments, higher *NCED3* transcripts in *35S:GmNFYA13* plants (Figures 4J, 5J) resulted in increased ABA biosynthesis and accumulation (Figures 4F, 5G), causing smaller stomatal apertures (Supplementary Figure 4) and less water loss in leaves (Figure 5C) to enhance tolerance to osmotic stresses. Moreover, *35S:GmNFYA13 Arabidopsis* seeds and seedlings displayed higher sensitivity to ABA compared with WT lines (Figure 3A and Supplementary Figure 3). Enhanced drought and salt tolerance was accompanied by ABA hypersensitivity (Ni et al., 2013; Chen et al., 2015; Ma et al., 2020). These results indicated that overexpression of *GmNFYA13* enhanced tolerance to osmotic stresses in an ABA-dependent manner. In addition, compared with corresponding lines with the normal *GmNFYA13* transcript, the higher expression levels of *DREB2A* and *GmDREB2* in *35S:GmNFYA13 Arabidopsis* and OE soybean lines (Figures 5H, 8A) showed that overexpression of *GmNFYA13* also enhanced osmotic stresses through another mechanism, an ABA-independent pathway.

The functional studies of *GmNFYA3* and *GmNFYB1* in transgenic *Arabidopsis* plants were professional (Ni et al., 2013; Li et al., 2017). However, the functions of these genes were determined in transgenic *Arabidopsis* plants. Introduction of SWP in this study (Figures 5B, 6B) during drought treatment was not performed in some other reports (Li et al., 2008; Chen et al., 2015; Li et al., 2017; Du et al., 2018; Li et al., 2019). Moreover, the genes in soybean should be identified in transgenic soybean plants.

Consistently, in comparison to EV soybean lines, the OE lines overexpressing *GmNFYA13* and RNAi lines suppressing it, respectively, displayed increased and decreased tolerance to osmotic stresses. The analysis of qRT-PCR (Figure 8) and LUC activity (Figure 9) indicated that GmNFYA13 could positively and directly regulate the transcript levels of downstream genes by binding to the promoters *in vivo*. *GmRbohB* was determined to be a key gene scavenging ROS to improve tolerance to abiotic stresses in soybean plants (Wang N. et al., 2017). Consistent with this result, transcript levels of *GmNFYA13* were induced by H_2_O_2_ solution (Figure 1C). In contrast to EV plants, the lower content of MDA (Figures 6E, 7F), which reflected the degree of the damage of ROS on membranes (Imahori et al., 2016; Wang X. Y. et al., 2017), was associated with the lower level of oxidative stress in OE transgenic soybean lines under salt and drought treatments. These results showed that overexpression of *GmNFYA13* enhanced tolerance to osmotic and oxidative stresses in OE transgenic soybean plants.

In conclusion, physiological indices and expression levels of stress-related genes shed light on the essential roles of multifunctional *GmNFYA13* and give further insights into the modulation of responses to osmotic stresses in soybean plants. Accordingly, *GmNFYA13* should be a positive regulator of tolerance to osmotic stresses in soybean and has application value to improve salt and drought tolerance in other plants.

## Data Availability Statement

Soybean gene sequences in this study were downloaded from the plant genomics resource (https://phytozome.jgi.doe.gov/pz/portal.html). *Arabidopsis* gene sequences in this study were downloaded from The *Arabidopsis* Information Resource (TAIR) (http://www.arabidopsis.org/). The gene accessions are listed in Supplementary Table 1.

## Author Contributions

Z-SX coordinated the project, conceived and designedexperiments, and edited the manuscript. X-JM performed experiments and wrote the first draft. J-DF and Y-MT conducted the bioinformatic work and performed experiments. T-FY, Z-GY, JC, Y-BZ, MC, and Y-ZM provided analytical tools and managed reagents, coordinated the project, and contributed valuable discussions. All authors have read and approved the final manuscript.

## Conflict of Interest

The authors declare that the research was conducted in the absence of any commercial or financial relationships that could be construed as a potential conflict of interest.

## Supplementary Material

Supplementary Figure 1 The synthetic fragment RNAi-*GmNFYA13*. The hairpin structure contained three parts: the positive sequence in blue, the reverse complementary sequence in green and the intron of GmNFYA13 in purple.

Supplementary Figure 2 The sequence alignment and phylogenetic analysis of *GmNFYA13* and ten NF-YA genes in *Arabidopsis*. **(A)** Sequence alignment of GmNFYA13 and ten NF-YA genes in *Arabidopsis* with conserved domains highlighted in black. The linker and two subdomains are underlined. Critical amino acids were indicated by asterisks. **(B)** Phylogenetic analysis of GmNFYA13 and ten NF-YA genes in *Arabidopsis*. MEGA 7.0 was used to construct the neighbor joining tree.

Supplementary Figure 3 Germination rate of WT and *35S:GmNFYA13* transgenic *Arabidopsis* lines under treatment of control, salt, drought and ABA. **(A)** Germination rates on 1/2 MS medium. **(B–C)** Germination rates on 1/2 MS medium with 50 and 80 mM NaCl. **(D–E)** Germination rates on 1/2 MS medium with 8 and 10% PEG6000. **(F–G)** Germination rates on 1/2 MS medium with 0.5 and 0.8 μM ABA. Asterisks represent significant differences at *P* < 0.05 in comparison to the corresponding controls. Each experiment had three biological replicates.

Supplementary Figure 4 Stomatal apertures of the leaves in WT and *35S:GmNFYA13* transgenic *Arabidopsis* lines were measured under control and ABA treatments. **(A)** Stomatal apertures subjected to 0 and 5 μM ABA solution. **(B)** The ruler tool developed in Adobe Photoshop CS5 was used to measure width/length of the stomatal aperture with 0, 5, and 10 μM ABA. Data indicate three biological replicates ± SD. Different letters above the columns represent significant differences at *P* < 0.05.

Supplementary Figure 5 The transcript levels of *GmNFYA13* in WT and *35S:GmNFYA13* lines were evaluated with RT-PCR. *AtTub8* was the internal control.

Supplementary Table 1 The primer sets and gene accessions for qRT-PCR used in this study.
