# Supplementary figures and images for: GmNFYA13 Improves Salt and Drought Tolerance in Transgenic Soybean Plants
Source: Front Plant Sci. 2020 Oct 23;11:587244. doi: 10.3389/fpls.2020.587244 (PMC7644530; doi:10.3389/fpls.2020.587244)

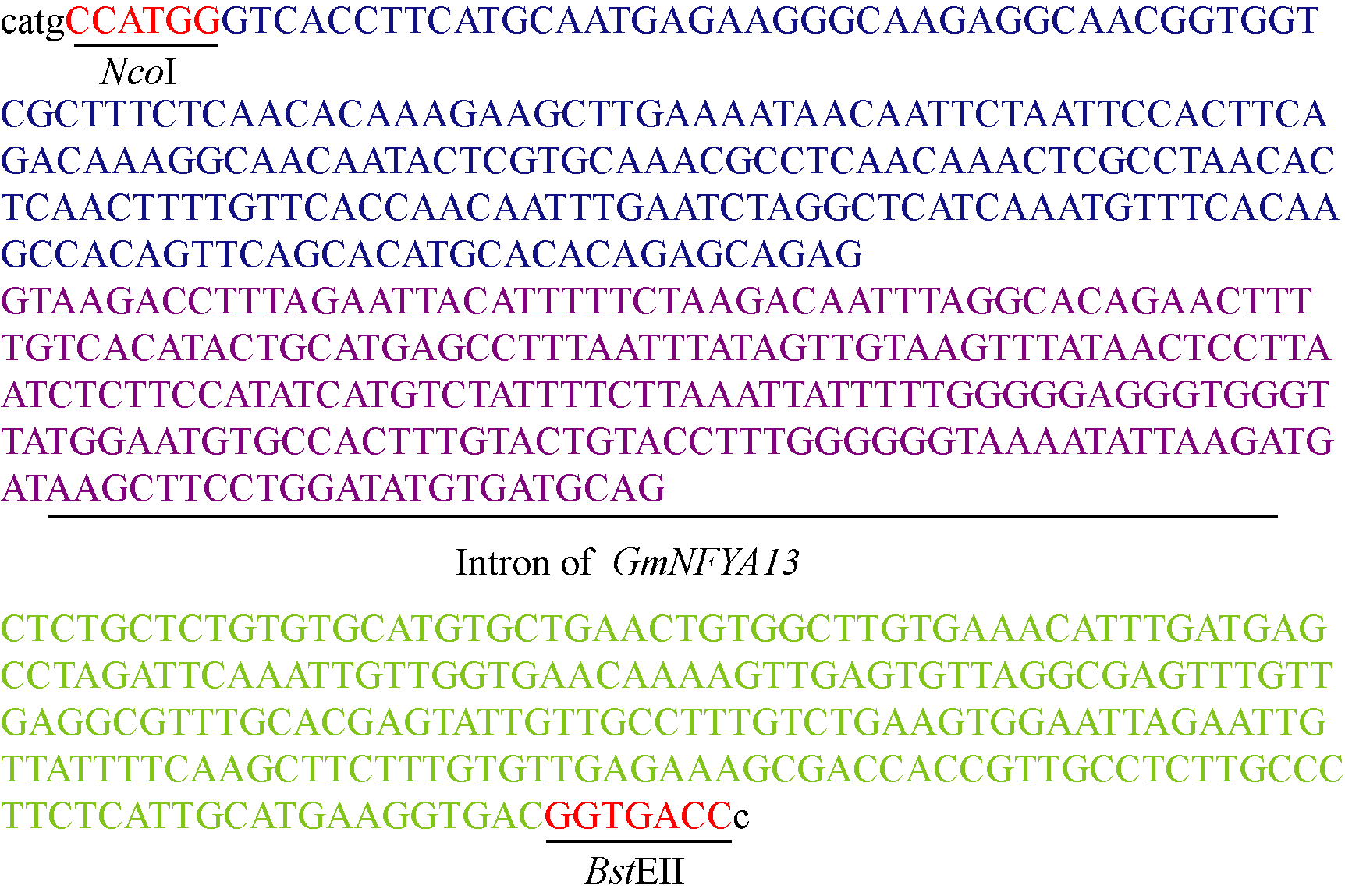

Supplement: Supplementary Figure 1 — The synthetic fragment RNAi-GmNFYA13. The hairpin structure contained three parts: the positive sequence in blue, the reverse complementary sequence in green and the intron of GmNFYA13 in purple. [file Image_1.TIF]

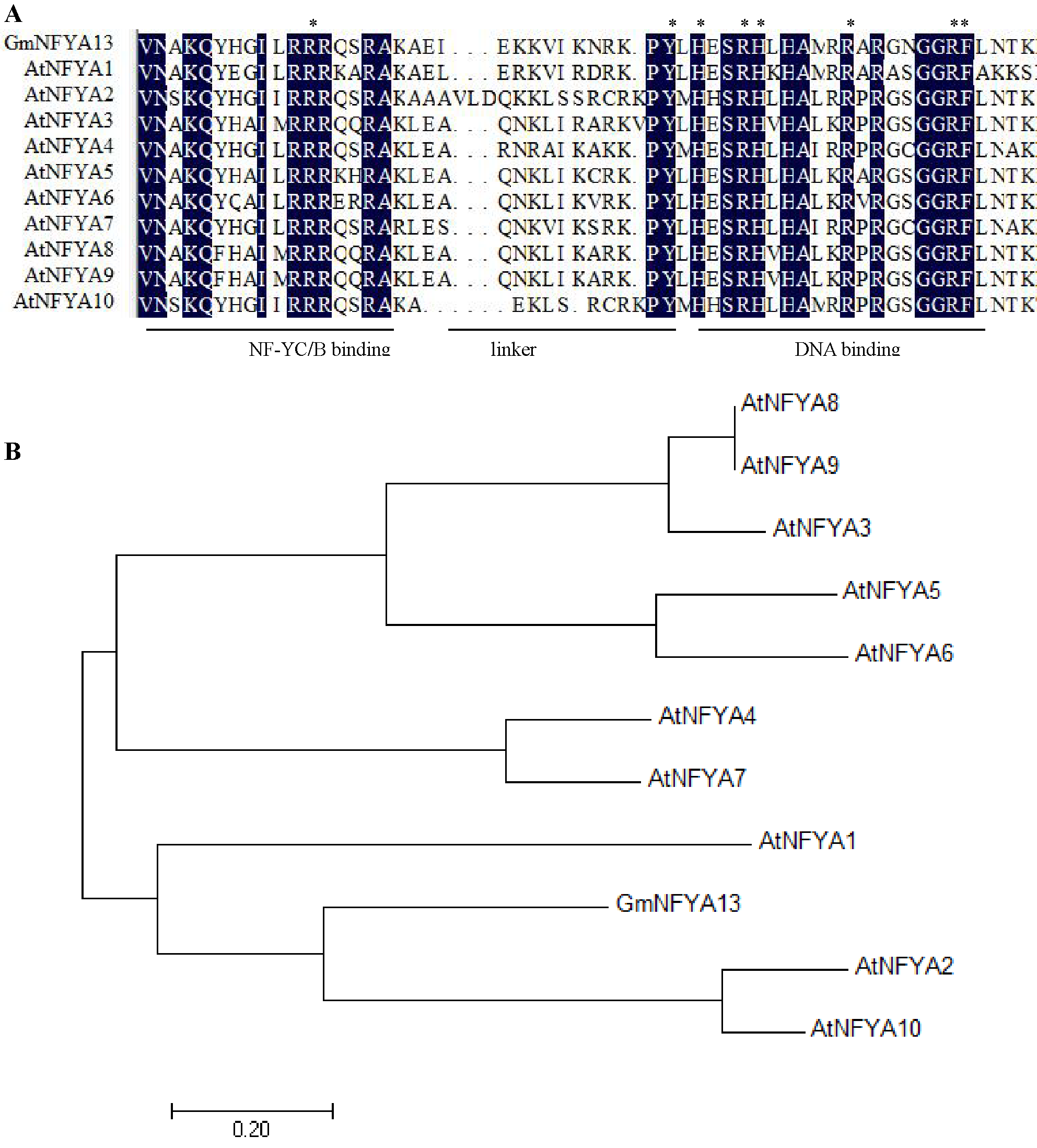

Supplement: Supplementary Figure 2 — The sequence alignment and phylogenetic analysis of GmNFYA13 and ten NF-YA genes in Arabidopsis. (A) Sequence alignment of GmNFYA13 and ten NF-YA genes in Arabidopsis with conserved domains highlighted in black. The linker and two subdomains are underlined. Critical amino acids were indicated by asterisks. (B) Phylogenetic analysis of GmNFYA13 and ten NF-YA genes in Arabidopsis. MEGA 7.0 was used to construct the neighbor joining tree. [file Image_2.TIF]

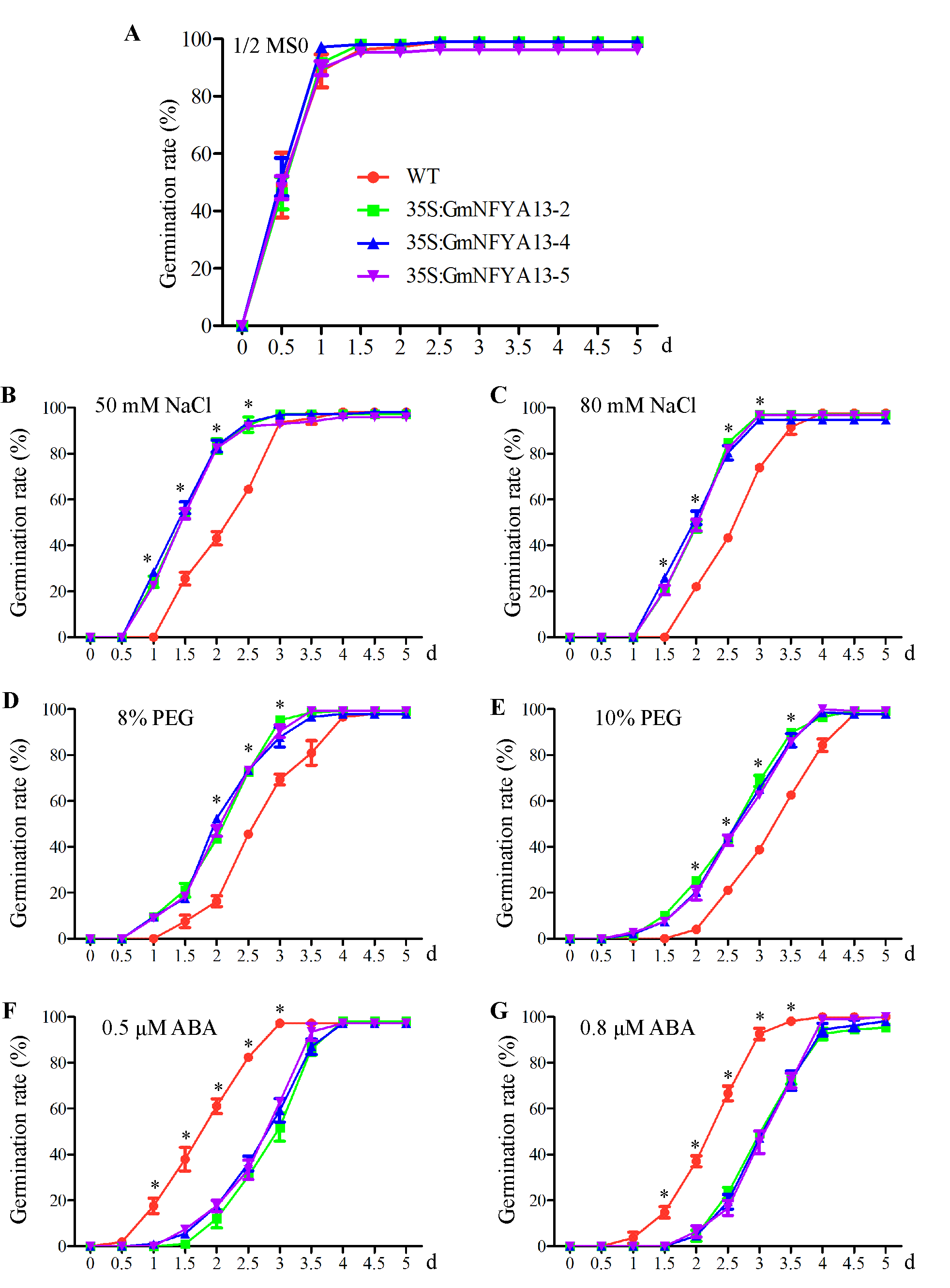

Supplement: Supplementary Figure 3 — Germination rate of WT and 35S:GmNFYA13 transgenic Arabidopsis lines under treatment of control, salt, drought and ABA. (A) Germination rates on 1/2 MS medium. (B–C) Germination rates on 1/2 MS medium with 50 and 80 mM NaCl. (D–E) Germination rates on 1/2 MS medium with 8 and 10% PEG6000. (F–G) Germination rates on 1/2 MS medium with 0.5 and 0.8 μM ABA. Asterisks represent significant differences at P < 0.05 in comparison to the corresponding controls. Each experiment had three biological replicates. [file Image_3.TIF]

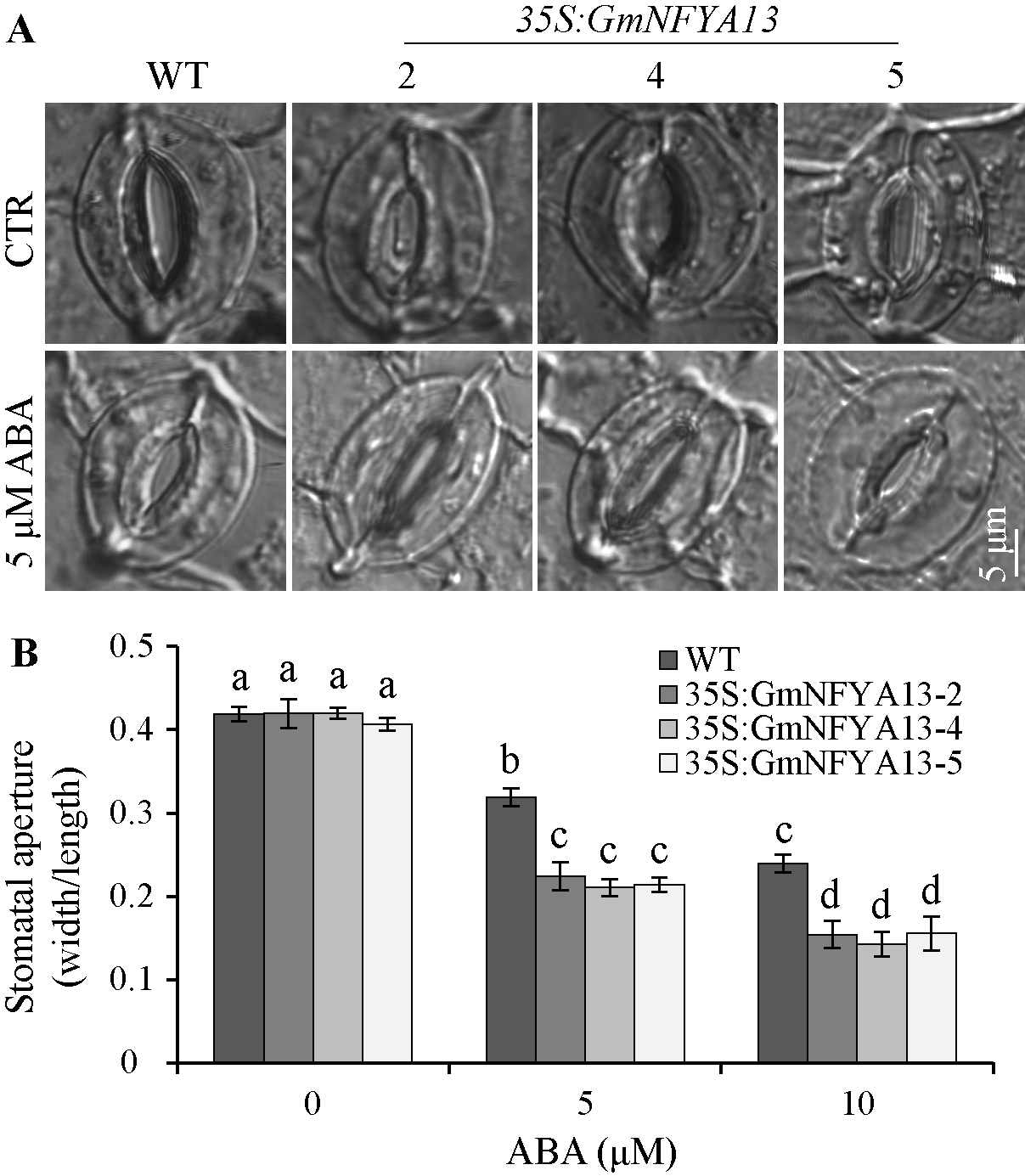

Supplement: Supplementary Figure 4 — Stomatal apertures of the leaves in WT and 35S:GmNFYA13 transgenic Arabidopsis lines were measured under control and ABA treatments. (A) Stomatal apertures subjected to 0 and 5 μM ABA solution. (B) The ruler tool developed in Adobe Photoshop CS5 was used to measure width/length of the stomatal aperture with 0, 5, and 10 μM ABA. Data indicate three biological replicates ± SD. Different letters above the columns represent significant differences at P < 0.05. [file Image_4.TIF]

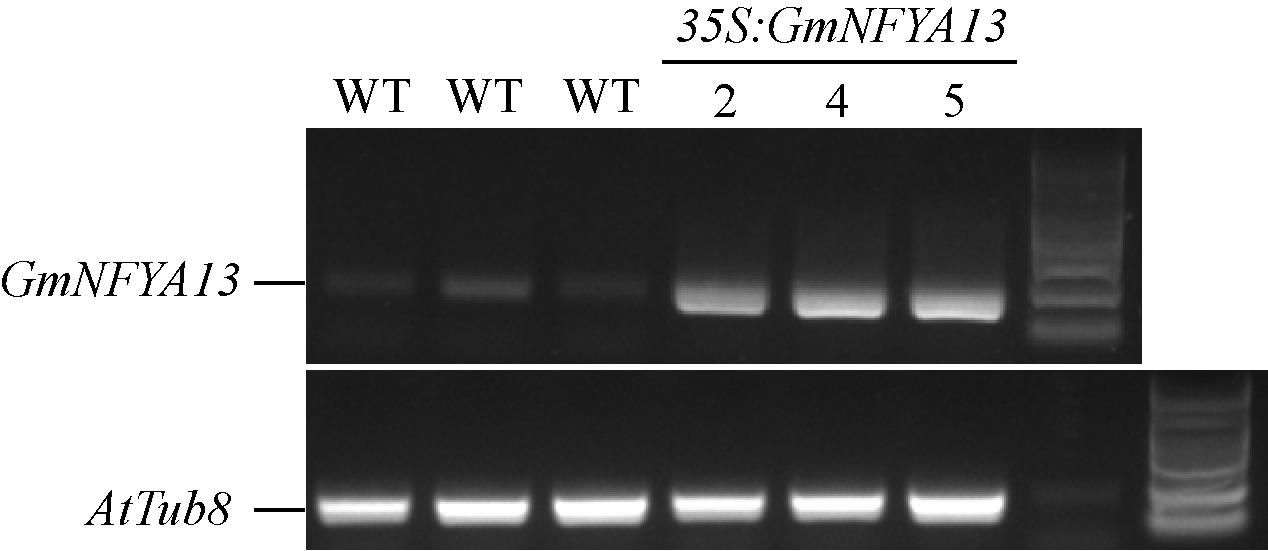

Supplement: Supplementary Figure 5 — The transcript levels of GmNFYA13 in WT and 35S:GmNFYA13 lines were evaluated with RT-PCR. AtTub8 was the internal control. [file Image_5.TIF]
